# Know your neighbors: microbial recognition at the intestinal barrier and its implications for gut homeostasis and inflammatory bowel disease

**DOI:** 10.3389/fcell.2023.1228283

**Published:** 2023-07-14

**Authors:** Krishna Iyer, Lena Erkert, Christoph Becker

**Affiliations:** ^1^ Department of Medicine 1, Universitätsklinikum Erlangen, Friedrich-Alexander-Universität Erlangen-Nürnberg, Erlangen, Germany; ^2^ Evergrande Center for Immunologic Diseases, Harvard Medical School and Brigham and Women’s Hospital, Boston, MA, United States

**Keywords:** intestinal epithelial cells (IECs), pattern recognition receptors (PRRs), mucosal immunity, intestinal homeostasis, microbiome, inflammatory bowel disease (IBD)

## Abstract

Intestinal epithelial cells (IECs) perform several physiological and metabolic functions at the epithelial barrier. IECs also play an important role in defining the overall immune functions at the mucosal region. Pattern recognition receptors (PRRs) on the cell surface and in other cellular compartments enable them to sense the presence of microbes and microbial products in the intestinal lumen. IECs are thus at the crossroads of mediating a bidirectional interaction between the microbial population and the immune cells present at the intestinal mucosa. This communication between the microbial population, the IECs and the underlying immune cells has a profound impact on the overall health of the host. In this review, we focus on the various PRRs present in different cellular compartments of IECs and discuss the recent developments in the understanding of their role in microbial recognition. Microbial recognition and signaling at the epithelial barrier have implications in the maintenance of intestinal homeostasis, epithelial barrier function, maintenance of commensals, and the overall tolerogenic function of PRRs in the gut mucosa. We also highlight the role of an aberrant microbial sensing at the epithelial barrier in the pathogenesis of inflammatory bowel disease (IBD) and the development of colorectal cancer.

## 1 Introduction

Intestinal epithelial cells (IECs) form a dynamic monolayer called the epithelial barrier, and together with the muscular layer, and connective tissue, make up the intestinal mucosa. A variety of absorptive and secretory cells, including the absorptive enterocytes, goblet cells, Paneth cells, tuft cells, and enteroendocrine cells, make up the intestinal epithelial layer. M or microfold cells contribute to an active microbial recognition and sampling in the intestinal lumen. These cells perform several metabolic and immune functions that are involved in maintaining intestinal homeostasis and overall epithelial barrier function. The intestinal epithelial layer is constantly exposed to a large population of microbes, both as part of the microbiota and potential pathogens, and pathobionts. IECs are thus at the crossroads of mediating a bidirectional interaction between the immune cells present at the mucosal layer, and the environment. However, to ensure a symbiotic relationship between the host and the indigenous commensal microorganisms, while allowing for efficient recognition and clearance of invading pathogens, microbial sensing at the mucosal surface of the gut must be tightly controlled (Shibolet and Podolsky, 2007). This is primarily achieved by a complex recognition system via pattern recognition receptors (PRRs), including the toll-like receptors (TLRs), NOD and NOD-like receptors (NLRs), RIG-I-like receptors (RLRs), C-type lectin receptors (CLRs), and Alpha-protein kinase 1 (ALPK1).

Microbial interaction with IECs also define several developmental and physiological functions in the host, and dysbiosis has been implicated in the pathogenesis of inflammatory bowel disease (IBD) and colorectal cancer (Tamboli et al., 2004; Carvalho et al., 2012). Of the trillions of microbes that occupy the habitat in close proximity to the epithelial barrier, both the microbial population and their interactions with different epithelial cells vary largely along the lengths of the small and the large intestine. PRR signaling, mediated by the MyD88 pathway in IECs, play a key role in controlling both the spatial segregation and composition of commensals. Secretion of mucus from the goblet cells in the colon and antimicrobial peptides from Paneth cells in the small intestine facilitate spatial segregation of commensals in the respective regions (Johansson et al., 2008; Vaishnava et al., 2011), suggesting that diverse mechanisms are involved in the establishment of an immunologically tolerated interaction between the IECs and the commensal microbes. Studies in germ-free mice demonstrate the beneficial effects of microbial colonization of the gut lumen on intestinal epithelial metabolism, proliferation, survival, barrier function, and on IEC communication with immune cells (Smith et al., 2007). This interaction at the epithelial barrier also promotes the development and the maturation of diverse immune cell populations residing in the underlying lymphoid tissues. Thus, the overall crosstalk between microbes, IECs, and the underlying immune cells define the immune responses in the region and shapes the overall metabolic and physiological processes in the host tissue.

## 2 Microbial recognition at the intestinal epithelial barrier

A number of somatically encoded PRRs are expressed in different cellular compartments of the IECs. The juxtaposition and varying expression patterns of these receptors long the small and the large intestine determine the microbial recognition and effector function of IECs at the epithelial barrier. Based on their location in the cell, PRRs can be broadly divided into two classes—those located on the cell membrane and those localized within cellular compartments such as the cytosol and endosomes.

### 2.1 Toll-like receptors, NOD and NOD-like receptors

Toll-like receptors (TLRs) are found on the cell surface as well as in cytosolic compartments like the endosomes. It has been reported that TLR expression varies significantly along the length of the small and large intestine (Price et al., 2018). *In-situ* and organoid-based studies of the differential expression patterns of TLRs reveal very low or no expression of TLR(s)- 2, −4, −5, −7, and −9 in IECs of the small intestine, while very high expression of TLR(s)- 2, −4, and −5 along colonic epithelial cells (Price et al., 2018). This differential pattern of expression of PRRs along the small and the large intestine defines an immunologically important effector function of epithelial cells in the context of their interaction with the microbial population. Classically, the majority of cell surface TLRs are involved in the recognition of bacterial surface structures such as lipopolysaccharide (LPS), lipoproteins, or flagellins. TLRs located in cytosolic compartments such as endosomes are involved in the recognition of nucleic acids such as microbial dsRNA, ssRNA, and dsDNA.

Nucleotide oligomerization domain (NOD)-like receptors (NLRs) and retinoic acid inducible gene-I (RIG-1)-like receptors (RLRs) are found in the cytosolic compartments of epithelial cells (Martinon and Tschopp, 2005; Thompson et al., 2011). NOD1 and NOD2 have been intensively studied in the gut and are responsible for recognition of bacterial cell wall peptidoglycan (PGN). NOD1 senses the meso-diaminopimelic type of PGN, which is most commonly found in Gram-negative bacteria (Girardin et al., 2003a; Girardin et al., 2003b). NOD2 has a broader sensing spectrum, recognizing the muramyl dipeptide N-acetylmuramyl-L-alanyl-D-glutamate, which is common to both Gram-negative and Gram-positive bacteria (Girardin et al., 2003b; Girardin et al., 2003c). NOD2 is highly expressed in Paneth cells of the small intestine and leads to cellular responses such as antimicrobial peptide (AMP) production, cytokine secretion, induction of autophagy, intracellular trafficking, and activation of epithelial regeneration (Couturier-Maillard et al., 2013; Nigro et al., 2014a; Ramanan et al., 2014). NOD1 and NOD2 have also been implicated in mediating beneficial interactions with the commensal flora (Eberl and Boneca, 2010).

**FIGURE 1:**
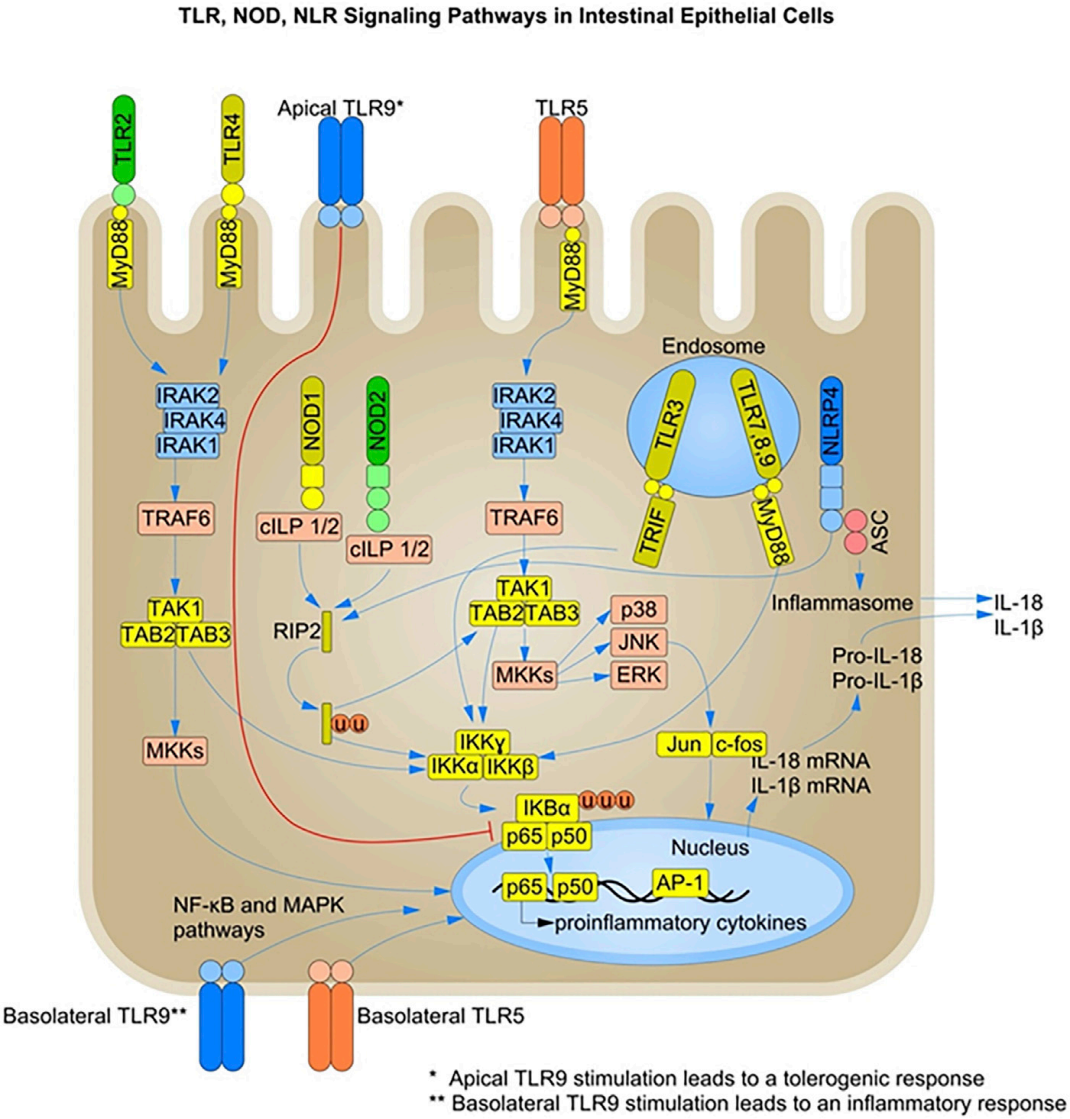
TLR-, NOD-, and NLR-mediated Signaling Pathways in Intestinal Epithelial Cells: Pattern recognition receptors (PRRs) such as toll-like receptors (TLRs) and nucleotide-binding oligomerization domain (NOD)-like receptors (NLRs) are present on intestinal epithelial cells (IECs). TLR recognition of MAMPs such as unmethylated CpG-containing DNA, flagellin, lipopolysaccharide (LPS) and lipoproteins, induces the recruitment of adaptor proteins like MyD88 and TRIF, leading to activation of the NF-κB and MAPK signaling pathways. NLRs such as NOD1 and NOD2 recognize bacterial peptidoglycans and activate the NF-κB and MAPK pathways through the recruitment of RIPK2. Members of the NLR family also initiate a pro-inflammatory response by activating the inflammasome complex and secreting active forms of IL-1β and IL-18. Under steady-state conditions, PRR stimulation in IECs leads to the production of AMPs and other mediators of gut homeostasis. Under inflammatory conditions, both surface and endosomal PRRs are stimulated, leading to a pro-inflammatory response and pathogen clearance. The basolateral and intracellular localization of PRRs is one of the mechanisms of immune response dampening and microbial tolerance at the epithelial barrier. TLR, Toll-like receptors; NOD, nucleotide-binding oligomerization domain; RIPK2, Receptor-interacting protein 2; MyD88, myeloid differentiation primary-response gene 88; MAPK, mitogen-activated protein kinase; NF-κB, nuclear factor-kB; NLR, nucleotide-binding oligomerization domain (NOD)-like receptors; TRIF, TIR-domain-containing adaptor protein inducing interferon-β; NLRP, NLR family pyrin domain-containing.

Activation of PRRs leads to a signaling cascade that triggers a transcriptional program, and many of these receptors share a common downstream signaling pathway. Nuclear factor kappa light chain enhancer of B cells (NF-κB) is known as a master transcription factor involved in immune signaling. In a resting state, it is sequestered in the cytosol. Activation of the NF-κB cascade leads to release of NF-κB from its inhibitors, resulting in nuclear translocation and transcription of genes. TLR signaling also activates MAPKs, which synergise with NF-κB to express cytokines, chemokines and antimicrobial effectors (Kagan et al., 2008) (Figure 1). TLRs, with the exception of TLR3, transmit signaling information through the recruitment of the adaptor molecule myeloid differentiation primary response gene 88 (MyD88). In contrast, TLR3 has been shown to induce IRF3 activation through the TRIF pathway. TLR4 and TLR5 are also involved in TRIF pathway signaling in IECs, activating IRF3 and type I interferon production. Notably, while TLR4 signaling via MyD88 not always requires plasma membrane trafficking to endocytic vesicles, it has been shown that TRIF-mediated signaling of TLR4 requires internalization of the receptor (Kagan et al., 2008). NOD1 and NOD2 also activate NF-κB through the receptor-interacting serine/threonine kinase (RIPK1) signaling pathway. NOD1 and NOD2 in the cytosol signal through MAPK and NF-κB and their activation requires both molecules to get recruited to the plasma membrane (Barnich et al., 2005; Lecine et al., 2007; Kufer et al., 2008). NF-κB activation by NOD1/2 requires the adaptor molecule RIPK2 (Park et al., 2007), whereas the MAPK pathway is mediated by CARD9 (Hsu et al., 2007).

NLRs are involved in the intracellular recognition and sensing of microbes or microbial products via the formation of molecular scaffold complexes called inflammasomes. Inflammasomes are macromolecular complexes that initiate an inflammatory response by activating caspase-1 in response to microbial recognition, cellular stress, or cellular damage (Von-Moltke et al., 2013). Inflammasome complexes contain a sensor protein from the nucleotide-binding domain and leucine-rich repeat protein (NLR) family, an adaptor protein such as apoptosis-associated speck-like protein containing a CARD (ASC), and caspase-1 (Figure 1). The activation of the inflammasome complex leads to the induction of inflammatory signals through the cleavage of pro-IL-1β and IL-18 into their active forms (Kamada et al., 2013).

Microbial recognition leads to the activation and expression of inflammasome components, and substrate cytokines in the infected cells. Several other cues, such as cellular insults like loss of membrane integrity due to pathogen invasion, can provide the second signal for activation, as in the case of the (NLR) family pyrin domain-containing 3 (NLRP3) inflammasome. This leads to caspase-1 cleavage and cytokine release. NLRP3 senses danger signals such as ATP release or potassium imbalance during infection (Petrilli et al., 2007). NLRP1 is expressed in glandular epithelial structures in the intestine (Kummer et al., 2007) and is involved in the detection of cellular toxins. The NLRP6-inflammasome is also highly expressed in IECs and drives mucus secretion from goblet cells by promoting autophagy (Wlodarska et al., 2014). However, the specific NLRs involved, the mechanism of activation of different inflammasomes and their exact role in intestinal epithelial function remain poorly understood (Cario, 2010).

**FIGURE 2:**
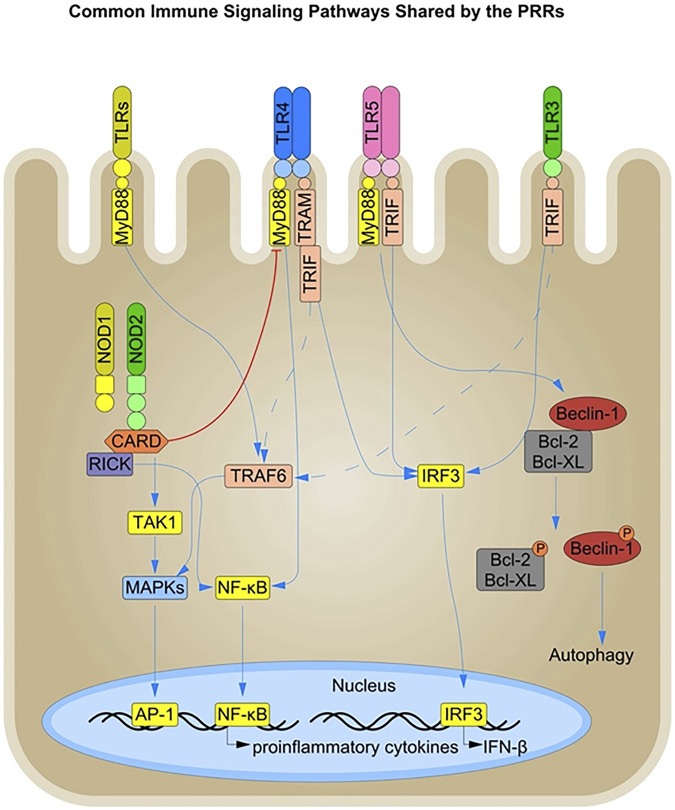
Immune Signaling Pathways shared by the PRRs in Intestinal Epithelial Cells: The PRRs present in the different cellular compartments of IECs, such as the cell surface, and endosomes, share a common downstream signaling pathway and thus have an overall complementary or inhibitory effect on each other. The TLRs signal downstream through the common MyD88, TRIF, and IRF3 signaling molecules. Signaling via the MyD88 and TRIF signaling components leads to nuclear translocation of NF-κB via the common TRAF6 molecule and production of pro-inflammatory cytokines. In addition, both TLR and NOD signaling converge to activate the MAPK pathway, amplifying the inflammatory response. Conversely, NOD2 activation is known to dampen TLR signaling such as TLR4 in certain inflammatory conditions. TLR signaling via the MyD88 and TRIF pathways also influences the induction of autophagy by interacting with Beclin-1, a key inducer of autophagosome formation. Beclin-1 directly interacts with the anti-apoptotic proteins Bcl-X_L_ and Bcl-2, and TLR activation leads to the phosphorylation and subsequent disassociation of Beclin-1 from Bcl-2 and Bcl-X_L_, resulting in the initiation of autophagy.

Autophagy is a crucial cellular defense mechanism involving microbial recognition and clearance by targeting them to the lysosomal compartment for degradation. Activation of NOD1 and NOD2 induces autophagy in response to pure microbe-associated molecular patterns (MAMPs) as well as bacterial infections such as *Listeria monocytogenes* and *Shigella* spp. in a RIPK2-independent manner (Travassos et al., 2010). TLR signaling also induces autophagy via the engagement of the adaptor molecule MyD88 and the TRIF pathway in macrophages and epithelial cells (Shi and Kehrl, 2008; Benjamin et al., 2013). Both MyD88 and the TRIF molecule interact with Beclin-1, the primary inducer of autophagosome formation, leading to its dissociation from the anti-apoptotic proteins Bcl-2 and Bcl-X_L_, thereby promoting autophagy (Figure 2) (Shi and Kehrl, 2008).

TLR and NLR activation lead to the recruitment of different adaptor molecules but result in similar downstream signaling with activation of NF-κB, MAPK, the inflammasome, and autophagosome formation. There is a dynamic crosstalk between the individual signaling pathways through the TLRs and NLRs that can be either synergistic or antagonistic in effect (Figure 2). For example, NOD2 activation in IECs has been shown to dampen TLR2 and TLR4 signaling, thereby preventing enhanced inflammation in the gut (Barreau et al., 2010). This suggests an interplay between PRRs present in different cellular compartments at the intestinal barrier, and an unequal division of the labor between them could explain the tolerogenic effect towards the commensal MAMPS at the epithelial barrier. However, the interplay between different PRRs has primarily been documented in immune cells (O’Neill, 2008) and their role in IECs needs further investigation (Rosenstiel et al., 2003).

### 2.2 C-type lectin receptors and RIG-1-like receptors

Members of the C-type lectin receptor (CLR) family recognize fungal pathogens and are also responsible for maintaining the fungal microbiota in the gut. Dectin1/2/3 and Mincle are the most notable members of the CLR family and are involved in the recognition of specific carbohydrate motifs on the fungal cell surface (Geijtenbeek and Gringhuis, 2016; Goyal et al., 2018). CLRs have been mainly characterized to be expressed on myeloid cells in the lamina propria, where the fungal molecules rarely come into direct contact during homeostasis (Volman et al., 2010). However, the role of CLRs in maintaining epithelial integrity and homeostasis cannot be undermined. Dysbiosis, in terms of loss of both bacterial and fungal populations from the gut, leads to the manifestation of IBD, and thus CLRs may play a critical role in microbial recognition and signaling even at steady-state (Belkaid and Hand, 2014; Francescone et al., 2014; Meng et al., 2018).

CLRs have been shown to exert a host-protective function in the context of dysbiosis. Dectin-2 knockout mice were found to be more susceptible to *Candida albicans* infections, an opportunistic pathobiont. This suggests a role for dectin-2 in recognizing the yeast and in suppressing the overgrowth of *C. albicans* (Ifrim et al., 2016). There are several factors that can lead to a breach in epithelial barrier integrity during different physiological states in the gut. A lack of, or over-activation of, certain immune responses due to a disturbance in the gut flora can damage the intestinal epithelial barrier, compromising the spatial separation between microbes and host tissue (Candela et al., 2014). This is one of the many plausible scenarios for the interaction of microbes with CLRs residing in the epithelial cells. Alternatively, microfold (M) cells serve as a portal for microbes to cross the barrier and induce subsequent immune responses in the lamina propria (Mabbott et al., 2013). CLRs are thought to play an important role in mediating pathogen recognition and immune response, and dectin-1 has been shown to play a role in defining the first line of defense in the gut. Dectin-1 pairs with Siglec-5 receptors and mediates the delivery of soluble immunoglobulin A (sIgA) via M cells (Rochereau et al., 2013). Members of the CLR family also perform immune functions by cooperating with other PRRs in the cell. Dectin-1 activation has been shown to act synergistically with TLR2, and TLR4 (Taylor et al., 2007; Ferwerda et al., 2008) in immune cells, and thus the possibility of similar associations in IECs may provide a basis by which CLRs mediate fungal recognition and signaling in epithelial cells.

Retinoic acid-inducible gene (RIG-I)-like receptors (RLRs) are a family of RNA helicases that recognize viral RNAs and induce innate antiviral responses via activation of pro-inflammatory cytokines and type-I interferon (IFN) (Rehwinkel and Reis-e Sousa, 2010). RLRs, RIG-I, and MDA-5 are RNA helicases containing a DExD/H box RNA helicase domain and two CARD-like domains and are located in the cytoplasm (Kawai and Akira, 2008). These protein domains can recognize viral RNA molecules and signal via NF-κB, MAPK, and IRFs. These translocate to the nucleus to promote transcription of genes encoding type I IFN and other proinflammatory cytokines. RIG-I, through the induction of an RNA polymerase III-transcribed RNA intermediate, has also been shown to sense AT-rich double-stranded DNA (Ablasser et al., 2009). However, the role of RLRs in viral DNA and RNA sensing and the effects of RLR-specific knockouts in viral microbiome maintenance and inflammation have only been studied in immune cells in the lamina propria, and their role in IECs requires further investigation. Furthermore, the ligands involved as well as the exact mechanism of activation of different RLRs like the RIG-I are not fully understood.

### 2.3 Alpha-protein kinase 1 (ALPK1)

**FIGURE 3:**
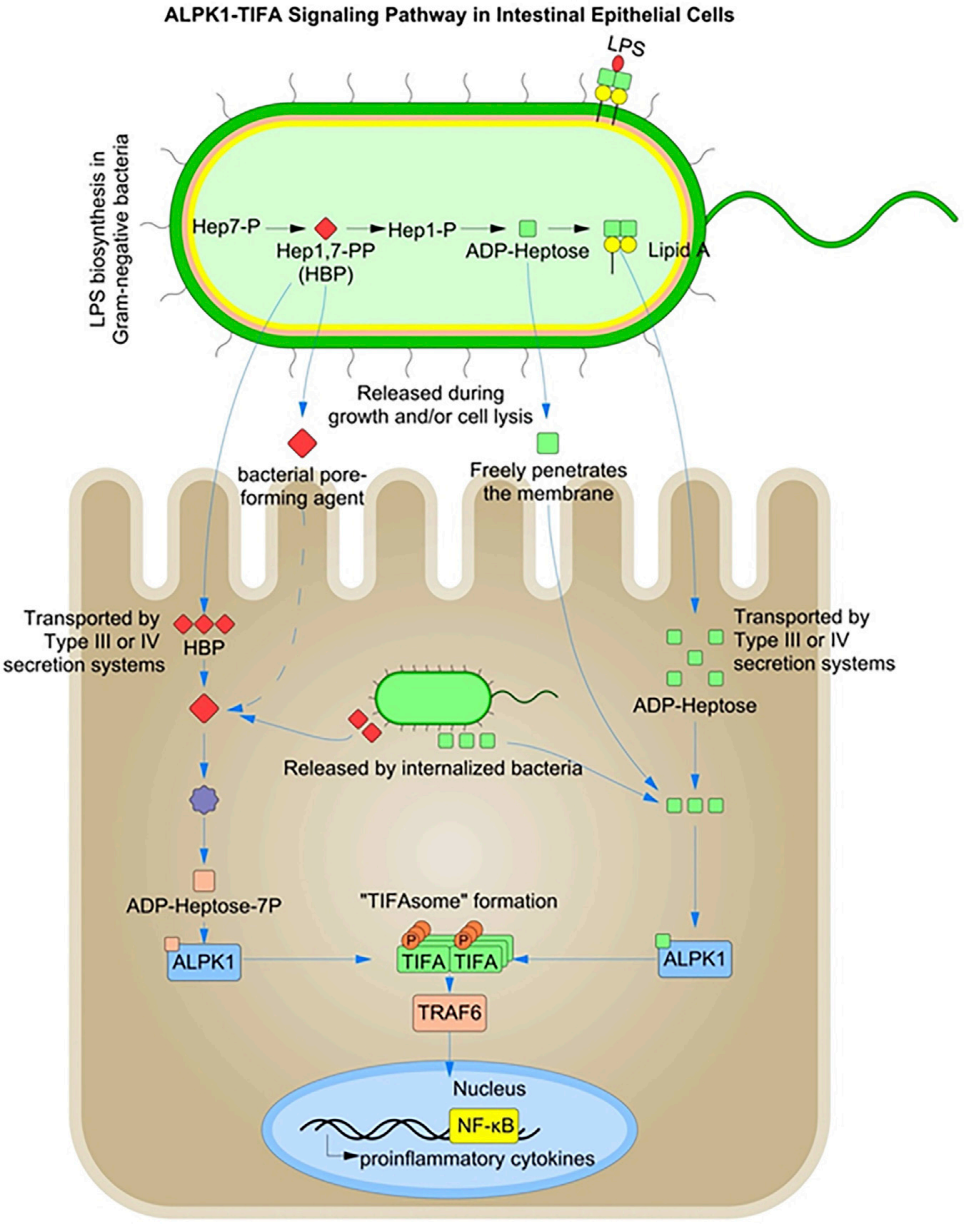
ALPK1-TIFA Signaling Pathway in Intestinal Epithelial Cells: Heptose 1,7-Bisphosphate (HBP) and ADP-β-d-manno-heptose (ADP-heptose) are intermediates in the biosynthesis of lipopolysaccharide (LPS). Both HBP, and ADP-heptose can activate a pro-inflammatory signaling pathway via ALPK1-dependent TIFA oligomerization in the cytosol.

The ALPK-1-TIFA-dependent cytosolic surveillance pathway is efficient at sensing the bacterial metabolites heptose-1,7-bisphosphate (HBP) and ADP-β-d-manno-heptose (ADP-heptose), both of which are an intermediate in bacterial LPS secretion (Gaudet et al., 2017; Pfannkuch et al., 2019) (Figure 3). HBP and ADP-heptose are produced by both pathogenic and commensal bacteria. A recent study showed that ADP-heptose has significantly higher activity than HBP and that cells are specifically able to detect the presence of the β-form, even when the compound is added extracellularly (Pfannkuch et al., 2019). The same study also found lower levels of HBP in *Helicobacter pylori* lysates, suggesting their inability to successfully activate the NF-κB pathway during infection and cytosolic invasion.

Pathogenic bacteria such as *Yersinia pseudotuberculosis* and *H. pylori* inject ADP-heptose into host cells using type III and IV secretion systems (Zimmermann et al., 2017; Zhou et al., 2018). In contrast, HBP recognition in the cytoplasm requires translocation by bacterial injection systems and must be enzymatically converted to ADP-heptose-7P to be detected by the cytoplasmic ALPK1-TIFA-associated surveillance system. However, ADP-heptose can freely cross the host cell membranes and enter the host cytoplasm (Xue and Man, 2018). HBP and ADP-heptose are metabolic intermediates in bacterial LPS biosynthesis and represent novel PAMPs, specific to Gram-negative bacteria (Gaudet et al., 2015). Host recognition of HBP requires its release from the bacterial cytosol by extracellular or intra-phagosomal bacteriolysis in case of enteric bacteria (Gaudet et al., 2015; Gaudet and Gray-Owen, 2016).

Thus, the alpha-protein kinase-1 (ALPK1) is primarily involved in the detection of freely replicating cytosolic bacteria and elicits a robust NF-κB response following activation of the peptidoglycan sensor NOD1 (Gaudet et al., 2017). NOD1 is known to mediate an initial transient burst of NF-κB activation during bacterial invasion (Girardin et al., 2003a). The ALPK1-TIFA-mediated pathogen recognition system is thought to play a role in supporting a sustained inflammatory response during the later stages of bacterial infection after the initial transient NOD1-mediated NF-κB activation.

Contamination of the cytosol with HBP/ADP-heptose has been shown to induce oligomerization of TIFA dependent on Thr-9 phosphorylation, which recruits and activates the E3 ubiquitin ligase TRAF6 to initiate a NF-κB-dependent pro-inflammatory transcriptional response. ALPK1 is responsible for TIFA oligomerization and IL-8 expression in response to the infection. The ALPK1-TIFA cytosolic surveillance pathway thus represents a NOD-independent mechanism for detecting invasive Gram-negative bacteria (Milivojevic et al., 2017). Intracellular infections, such as *Shigella flexneri,* can also be detected indirectly via damage-associated molecular patterns (DAMPs). Membrane vacuolar remnants produced after vacuolar lysis have been shown to be detected by host cells and the signals produced contribute to enhanced inflammation (Dupont et al., 2009). Accumulation of diacylglycerol around the bacterial entry site and within membrane remnants has been shown to activate NF-κB via a mechanism dependent on the CARD–BCL10–MALT1 complex and TRAF6 (Sanada et al., 2012).

The exact mechanism by which HBP and/or ADP-heptose induces TIFA-dependent activation in both infected and bystander cells is not fully understood. Gaudet *et al.* demonstrated IL-8 production in response to *S. flexneri* and *Salmonella typhimurium* infection due to HBP endocytosis (Gaudet et al., 2015). However, studies by Kasper *et al.* have shown that non-invasive *S. flexneri* bacteria do not induce an IL-8 expression (Kasper et al., 2010). Studies by Lippmann *et al.* also show that IL-8 expression in bystander cells requires bacterial internalization and that mere diffusion of HBP does not lead to TIFA activation (Lippmann et al., 2015).

Therefore, a more detailed investigation of intracellular HBP/ADP-heptose detection and its ability to induce TIFA activation is warranted. ADP-heptose can be classified as a small-diffusing molecule, but its role as a potent PAMP for PRR activation depends on its ability to serve as a marker of microbial invasion and cytosolic proliferation.

### 2.4 Other receptors involved in microbial recognition

There are other receptors on IECs that are involved in the detection of various microbial components and metabolites, and their role is of paramount importance in both defense and maintenance of intestinal homeostasis.

IECs are actively involved in the sensing of microbial metabolites produced by bacterial fermentation of dietary components in the gut, which may be particularly important for establishing a symbiotic relationship with the commensals and thereby defining a number of physiological roles in the gut. The pregnane X receptor (PXR) (Venkatesh et al., 2014) and the aryl hydrocarbon receptor (AhR) (Zelante et al., 2013; Metidji et al., 2018) are involved in the sensing of tryptophan catabolites produced by microbes in the lumen. PXR is sensitive to indole-3-propionic acid, a metabolite of tryptophan that is produced by the commensal *Clostridium* sporogenes. PXR knockout mice have increased inflammatory damage to the epithelium and decreased expression of the tight junction protein (TJP) (Venkatesh et al., 2014), supporting its role in a variety of anti-inflammatory and protective barrier functions in the gut.

IECs infected with *Yersinia enterocolitica* have been shown to use β1 integrins as Pathogen Recognition Receptor that recognize the bacterial adhesin called invasin. The invasin-integrin interaction provides an initial signal for activation of the NLRP3 inflammasome (Thinwa et al., 2014). Hydroxycarboxylic acid receptor-2 (GPCR-109A) is involved in the sensing of butyrate and niacin. Together with AIM-2 (Singh et al., 2014; Macia et al., 2015) and NLRP3, hydroxycarboxylic acid receptor-2 is involved in fine-tuning of IL-18 levels in the intestine. Epithelial IL-18 plays a central role in orchestrating the intestinal host–microbial homeostasis, and genetic deletion of these receptors results in intestinal inflammation, tumorigenesis, and increased susceptibility to enteric infections (Song-Zhao et al., 2014; Man et al., 2015).

More recently, the role of tuft cells in eliciting type 2-mediated immunity to allergens, helminth and protist infestations has been linked to their chemosensory capabilities in the small intestine. Tuft cells are a part of an elaborate tuft type 2 innate lymphoid cell (ILC-2) network and are critical for the activation of ILC-2 cells through the secretion of IL-25 and its downstream adaptor Act-1 (Kang et al., 2012). Tuft cells are thought to have distinct sensing mechanisms for both helminth and protist infestations with specific enrichment in G protein-coupled sensory receptor(s) and transmit downstream signals to activate type 2 immune cells (Nadjsombati et al., 2018). The extracellular succinate receptor (SUCNR1) has been identified to be expressed in both IL-25+ and TRPM5+ tuft cells in the small intestine (Bezencon et al., 2008; Lei et al., 2018; Nadjsombati et al., 2018), and succinate has been shown to act as an innate immune ligand sufficient to activate type 2 inflammation in mice. Furthermore, tuft cells also express other metabolic sensing receptors such as free fatty acid receptor 3 (FFAR3) (Schneider et al., 2018), but their role in orchestrating the type 2-mediated immune response is not fully understood. Tuft cells also express enzymes involved in the biosynthesis of eicosanoids, such as 5-lipoxygenase (Alox5), Cox-1, Cox-2, and hematopoietic PG-D synthase (HPGDS), and proliferate in an inflammatory environment (Gerbe et al., 2011; Von-Moltke et al., 2016).

## 3 Impact of microbial recognition at the intestinal epithelial barrier

### 3.1 Barrier function and maintenance of intestinal homeostasis

Upon detection of microbial patterns in the intestinal lumen, IECs enhance intestinal barrier functions, including mucus and AMP production, improved tight junction integrity, and mediating cell proliferation and differentiation to protect the bowel wall from microbial infiltration. We summarize different PRR mediated downstream signaling in cells on the epithelial barrier and their role in mediating either a pro- or anti-inflammatory response in Table 1.

**TABLE 1:** Pathogen Recognition Receptors (PRRs) expressed on different cells at the intestinal epithelial barrier and their downstream signaling effect in mediating a pro- or anti-inflammatory response in IECs.

Pathogen Recognition Receptor (PRR)	Expressed in IEC cell-type	PRR activation results in	Pro/Anti-inflammatory	References
Toll-like Receptors (TLRs)				
TLR-1	Enteroendocrine cells	NF-icB and MAPK activation; TNF-a expression	Pro-inflammatory	Bogunovic et al. (2007)
TLR-2	Enteroendocrine cells	NF-icB and MAPK activation; TNF-a expression	Pro-inflammatory	Bogunovic et al. (2007)
TLR-3	Paneth cells	Paneth cell degranulation	Anti-inflammatory	Rumio et al. (2011)
TLR-4	Enteroendocrine cells	NF-icB and MAPK activation; TNF-a expression Chemokine induction	Pro-inflammatory	Bogunovic et al. (2007); Selleri et al. (2008)
	Paneth cells	Paneth Cell Degranulation	Anti-inflammatory	Rumio et al. (2011)
TLR-5	Enteroendocrine cells	Chemokine induction	Pro-inflammatory	Selleri et al. (2008)
	Paneth cells	Paneth cell degranulation	Anti-inflammatory	Rumio et al. (2011)
		Chemokine and cytokine production	Pro-inflammatory	Price et al. (2018)
TLR-9	Enteroendocrine cells	Secretion of cholecystokinin	Anti-inflammatory	Daly et al. (2020)
NOD and NOD-like receptors (NLRs)				
NOD-1	Paneth Cells	NF-icB and MAPK activation; TNF-a expression Paneth Cell degranulation; induction of autophagy	Pro-inflammatory	Barnich et al. (2005), Lecine et al. (2007), Kufer et al. (2008)
NOD-2	Paneth Cells	NF-icB and MAPK activation; TNF-a expression Paneth Cell degranulation; induction of autophagy	Pro-inflammatory	Couturier-Maillard et al. (2013); Nigro et al. (2014a); Ramanan et al. (2014); Barnich et al. (2005); Lecine et al. (2007); Kufer et al. (2008)
NLRP3	Paneth Cells	Paneth Cell degranulation	Pro-inflammatory	Yokoi et al. (2019)
NLRP6	Goblet Cells	Inflammasome activation; Induction of autophagy leading to mucus secretions	Anti/Pro inflammatory	Cario (2010)
Other Receptors on IECs				
SUCNR1	Tuft Cells	Induction of type-2 inflammatory reaction	Pro-inflammatory	Bezencon et al. (2008); Lei et al. (2018); Nadjsombati et al. (2018)

IECs secrete a variety of AMPs through PRR/MyD88-dependent mechanisms (Vaishnava et al., 2008), which accumulate in the mucus layer and exert antimicrobial activities (Fahlgren et al., 2003). Paneth cells are involved in the secretion of α-defensins such as HD-5/6 in humans and cryptidins and CRS in mice. They also secrete other AMPs like RegIIIα/β/γ, sPLA2, and lysozyme-C in humans and mice (Brandl et al., 2007; Rakoff-Nahoum and Medzhitov, 2007; Gong et al., 2010). Enterocytes are primarily involved in the secretion of β-defensins in both humans and mice (O’Neil et al., 1999; Simmons et al., 2002). During an infection with *Citrobacter rodentium*, MyD88 signaling in IECs alone was found to be sufficient to improve epithelial barrier integrity and to increase production of RegIII-γ and the acute phase protein serum amyloid A1 (SAA1) (Friedrich et al., 2017).

Goblet cells form a viscous layer of mucus on the epithelial surface by secreting mucin glycoproteins. A discontinuous mucus layer in the mouse cecum and corresponding areas of the epithelium have been shown to form hotspots for microbial infection (Furter et al., 2019). Recognition of microbial LPS by LPS binding protein (LBP) and TLR4 elicits a pro-inflammatory response that induces expression of mucins in goblet cells (Smirnova et al., 2003). Inflammasome-mediated activation of NLRP6 has also been implicated in goblet cell mucus secretion through the promotion of autophagy (Cario, 2010).

The interaction of PRRs with commensal MAMPs also has a positive effect on mucus production by goblet cells at the epithelial barrier under homeostatic conditions. Amuc-1100 is a membrane protein of the commensal *Akkermansia muciniphila* that actively interacts with TLR2, resulting in increased mucus thickness and TJP expression at the epithelial barrier (Plovier et al., 2017). Microbiota-derived short-chain fatty acids (SCFAs) have been shown to regulate a number of IEC functions, including cell turnover (Park et al., 2016), TJP expression (Zheng et al., 2017), and upregulation of inflammasome- or hypoxia-inducible factor (HIF)-mediated epithelial integrity (Kelly et al., 2015; Macia et al., 2015).

Bacterial LPS can directly stimulate Paneth cells and apical stimulation of TLRs-2/3/4, NOD1/2 and NLRP3 leads to secretion of AMPs via immediate degranulation (Yokoi et al., 2019). MyD88-deficient mouse models have shown decreased production of RegIIIγ, RELMβ, and RegIIIβ in the intestinal epithelium (Gong et al., 2010), suggesting a correlation between PRR stimulation and AMP secretion. Paneth cells also interact with both pathogenic and commensal microbes in an alternative, indirect manner through the release of pro-inflammatory IFN-γ (Farin et al., 2014; Burger et al., 2018) to further secrete antimicrobial peptides.

The production of mucus and AMPs such as RegIIIγ in response to the microbial recognition by epithelial cells is critical for maintaining overall immune homeostasis and defining the spatial segregation between the host tissue and the commensals (Vaishnava et al., 2011). MyD88-dependent TLR signaling has been shown to be critical for protection against mucosal damage (Fukata et al., 2005; Choi et al., 2010). TLR2 signaling in IECs induce the expression of the TJP ZO-1, strengthening the epithelial barrier integrity and providing resistance to apoptosis (Cario et al., 2004; Cario et al., 2007). TLR2 signaling also induces the production of cytoprotective trefoil factor, involved in mucosal tissue repair (Podolsky et al., 2009). TLR4-mediated signaling in IECs via MyD88 further induces cyclooxygenase 2 (COX-2), which enhances prostaglandin E2 (PGE2) synthesis, thereby promoting epithelial cell survival (Pull et al., 2005; Fukata et al., 2006; Brown et al., 2007; Hernandez et al., 2010). TLR4 signaling in IECs has further been shown to induce the secretion of amphiregulin and epiregulin, which activate epidermal growth factor (EGF) receptors (Fukata et al., 2007).

Intestinal stem cells (ISCs) located at the bottom of crypts of Lieberkühn play a critical role in the maintenance of epithelial barrier integrity due to their ability to propagate progeny that differentiate into diverse cell-types depending on the physiological demands. The Lgr5+ ISCs are known to express PRRs, in particular TLR4 and NOD2. These play important roles in the maintenance of intestinal homeostasis by effecting stem cell survival, proliferation, and apoptosis (Nigro et al., 2014b). TLR4 signaling has been demonstrated to affect ISC proliferation and differentiation by influencing Wnt and Notch signaling in the intestinal crypts (Sodhi et al., 2011; Sodhi et al., 2012). Stem cells are key to warrant repopulation of the intestinal epithelium during the resolution phase of inflammation when the inflammation-induced damage has to be repaired. Although stem cells reside deep within the crypts in an area considered largely inaccessible for microbes in healthy individuals, in the inflamed gut with epithelial erosions and barrier dysfunction, TLR-mediated signaling in stem cells driving proliferation and inhibiting cell death becomes highly relevant for tissue recovery.

TLR signaling in Paneth cells has been shown to regulate the release of antimicrobial peptides and to play an important functional role in host defense and in the maintenance of gut homeostasis. For example, Rumio and others showed that engagement of TLR9, using the agonist CpG oligodeoxynucleotide (ODN) *in vivo,* leads to Paneth cell degranulation. Similarly, the TLR3 agonist polyinosinic-polycytidylic acid induced a strong and rapid degranulation, whereas the TLR4 agonist LPS and the TLR5 agonist flagellin induced only a late degranulation of Paneth cells (Rumio et al., 2011). Interestingly, a recent study by Price et al. examined TLR expression along the intestine and villus-crypt axis and showed that TLR5 expression in the small intestine is restricted to the Paneth cells, suggesting that TLR5 is particularly important for microbial sensing via Paneth cells. Remarkably, in this study, TLR5 signaling in Paneth cells did not induce antimicrobial peptides itself, but rather elicited chemokine and cytokine responses via Ccl20 and TNF-α, as well as NF-κB pathway-related molecules, including A20, Iκbα and Nfκb2. Antimicrobial peptide production was then shown to be indirectly induced by these inflammatory cytokines (Price et al., 2018). Paneth cells not only express TLR, but also express NLRs such as NOD2 (Lala et al., 2003) and NOD2^*−/−*^ mice have reduced Paneth cell-related α-defensin transcripts, including cryptidin-4 and cryptidin-10, and show increased susceptibility to infection when challenged with *Listeria monocytogenes* (Kobayashi et al., 2005).

Enteroendocrine cells also express several functional TLRs, including TLR1, TLR2, and TLR4, ultimately leading to NF-κB and MAPK activation and TNF-α expression (Bogunovic et al., 2007). Interestingly, a study by Selleri et al. showed that the TLR agonists LPS and flagellin are able to induce pro-inflammatory chemokines, such as CXCL1 and IL-32 specifically within enteroendocrine cells, suggesting this rare cell type as an important contributor in inflammatory processes in the gut (Selleri et al., 2008). More recently, TLR9 has been shown to be specifically expressed by enteroendocrine cells of the proximal intestine, where it leads to the secretion of cholecystokinin upon stimulation, which could ultimately lead to the elimination of pathogens through cholecystokinin-stimulated emesis, demonstrating a critical role for enteroendocrine cells in enteric infections (Daly et al., 2020). Altogether, these data demonstrate multiple layers of PRR-mediated interaction between the microbiota and the gut epithelium with its diverse cell types. Signaling via different PRR located in different compartments on the cell surface but also within the cell and its organelles allows epithelial cells to detect the nature of the microbial signal and the potential challenge it might pose for host defense. Receptor activation induces signaling cascades that regulate diverse epithelial cell functions, including proliferation and cell death, barrier integrity, metabolism and innate immunity.

### 3.2 Impact on the diversity of the gut microbiota

The presence of PRRs in different cellular compartments of the IECs has a profound effect on the overall maintenance of the commensal microbial population at the intestinal barrier. In exchange for this symbiotic relationship, several factors produced by the microbial population shape the overall immune system at the mucosal region and influence the various developmental and metabolic processes in the host tissue.

Metabolites of certain commensal spore-forming bacteria such as *Clostridium spp*. are known to promote serotonin (5-hydroxytryptamine (5-HT)) secretion from a subtype of enteroendocrine cells called enterochromaffin cells in colonized mice (Yano et al., 2015). The neurotransmitter serotonin is an important regulator of enteric nervous system development, gastrointestinal tract motility, and inflammation (Terry and Margolis, 2017). Microbial sensing by TLRs present on enteroendocrine cells also promotes the secretion of several other hormones such, as glucagon-like peptide 1 (GLP-1) (Lebrun et al., 2017) and peptide tyrosine-tyrosine (PYY) (Larraufie et al., 2017). Collectively, these hormones increase insulin secretion (Grøndahl et al., 2017), regulate mood, and induce satiety (Loh et al., 2015), thereby influencing overall host physiology.

Stimulation of the innate immune response by the microbiota also provides indirect resistance to infection in the gut. The depletion of commensal microbes in the gut has a direct effect on the viral immunity (Ichinohe et al., 2011; Abt et al., 2012). Induction of IFN-λ and IL-18 or IL-22 is essential for an effective antiviral innate immunity in the gut (Zhang et al., 2014; Nice et al., 2015). Commensals stimulate the production of IL-18 and IL-22, but actively suppress IFN-λ production, promoting viral persistence as a part of the gut microbial population (Baldridge et al., 2015). However, dysbiosis can lead to an imbalance in this complex loop, resulting in pronounced antiviral immunity in the gut.

Alterations in the normal resident microbial flora alter the normal gut immune response, as has been shown in the case of altered antimicrobial response following antibiotic treatment (Cash et al., 2006). As the production of AMPs requires TLR-dependent stimulation of Paneth cells, an imbalance in the microbiota population has been associated with impaired resistance to bacterial infections (Brandl et al., 2007; Brandl et al., 2008). MyD88-dependent TLR signaling at the intestinal barrier is also essential for maintaining the spatial segregation of commensal microbes and host tissues (Vaishnava et al., 2011). MyD88-mediated secretion of RegIIIγ anti-bacterial lectin has been shown to define both the composition and the spatial localization of the intestinal microbiome. This could fundamentally determine the balance between tolerogenic and pro-inflammatory immune responses in the gut. In the absence of MyD88, commensal bacteria have been shown to gain proximity to the intestinal surface, resulting in a manifold increase in mucosa-associated bacteria compared to that in wild type mice (Vaishnava et al., 2011).

PRR signaling also plays a pivotal role in defining the diversity of the commensal population at the gut. This has been suggested by the occurrence of dysbiosis (Tamboli et al., 2004), its association with polymorphisms in the gene encoding for NOD2, and its overall impact on host susceptibility to IBD such as Crohn’s disease (Hugot et al., 2001; Ogura et al., 2001). Thus, the antimicrobial peptides and secretory IgA produced in response to microbial sensing, balance the microbial composition, thereby limiting the penetration of commensal bacteria into the gut (Macpherson and Uhr, 2004). This regulation of bacterial load and composition may be one of the primary functions of PRRs in maintaining intestinal homeostasis.

### 3.3 IEC-microbiota-immune cells cross-talk

The signaling at the epithelial layer is not limited to the barrier region, but the resulting effector molecules are actively disseminated to the underlying mucosal layer. This, in turn influences the development and maturation of the underlying immune cells.

The presence and function of M-cells at the epithelial barrier suggests a much more dynamic role for epithelial cells in microbial sensing and sampling than that of a rigid barrier system. M cells are specialized cells that mediate a direct uptake of antigens and intact microbes from the intestinal lumen and transport them for presentation to resident immune cells. This effectively activates the adaptive immune system. M-cells initiate phagocytosis of the pathogen at the intestinal barrier upon recognition via PRRs. GP2 functions as a receptor for type I pili on a subset of Gram-negative enterobacilli (Hase et al., 2009) and is essential for immune surveillance at mucosal surfaces. Cellular prion protein (PrPc) is a glycosylphosphatidylinositol (GPI)-anchored protein that is expressed on the apical surface of M cells (Nakato et al., 2009). PrPc interacts with pathogens that contain heat shock protein 60 (HSP-60), a conserved surface protein with immunogenic properties (Kaufmann, 1990).

Specific microbial signaling at the epithelial barrier also plays a role in immune cell development. In particular, epithelial NOD1 signaling has been shown to be important for C-C motif chemokine 20 (CCL20)-mediated generation of isolated lymphoid follicles from cryptopatches (CPs) in the gut (Bouskra et al., 2008). Likewise, signaling via the innate receptors present on myeloid cells of the lamina propria also affects epithelial cells and the microbial population further in the intestinal lumen. Myeloid cells modulate key pathways such as IL-22 cytokine expression by innate lymphoid cells (ILCs) and induce the production of the antimicrobial peptides RegIIIβ and RegIIIγ, which are important for maintaining a spatial separation between the commensals and the intestinal epithelial layer. This modulation plays an integral role in supporting the spatial separation between commensals and the intestinal mucosa (Zheng et al., 2008; Vaishnava et al., 2011; Sonnenberg et al., 2012).

Another direct consequence of microbial sensing at the epithelial layer is related to the secretion of immunoglobulin A (IgA) in the gut. IgA has a specific role in the mucosal immune system and also plays an important role in maintaining the spatial segregation and composition of luminal microorganisms. TLR signaling in IECs induces expression of B cell activating factors that induce immunoglobulin class switch recombination in lamina propria B cells in a T cell-independent manner (He et al., 2007; Shang et al., 2008). TLR signaling in IECs also results in the secretion of April and BAFF. April directly induces IgA class switching recombination, while BAFF promotes B cell proliferation and survival. Furthermore, IECs can also indirectly induce class switching recombination by secretion of TSLP, which stimulates dendritic cells in the lamina propria to secrete April (He et al., 2007).

Activation of TLR3, and TLR4 has also been shown to induce the expression of polymeric immunoglobulin receptors involved in the epithelial transport of immunoglobulin, thereby enhancing the luminal IgA secretion (Schneeman et al., 2005). Thus, TLR signaling in IECs is actively involved in multiple steps of intestinal IgA secretion.

### 3.4 Tolerogenic effect of microbial recognition at the epithelial barrier

A very important aspect of the overall immune function at the intestinal mucosa is the ability of both the epithelial and immune cells to discriminate microbial cues from commensals as opposed to invasive pathogens. This is critical for maintaining a symbiotic relationship between the host and the microbial population present at the epithelial barrier. The successful establishment of the microbial niches along the elaborate spaces of the epithelial barrier is the result of the selective tolerogenic effect of the immune system towards commensal microbes. A number of topological, metabolic and genetic factors play an important role in establishing a fine line between the tolerogenic and defensive functions of the immune system.

The juxtaposition and polarity of PRR expression on epithelial cells play a very primitive but consequential role in determining the tolerogenic effect at the epithelial barrier. The unequal division of labor between the PRRs present on the epithelial cell surface and those in the cytosolic cellular compartments also provides a basis for tolerance. Only invasive microbes that manage to penetrate the barrier are detected by cytosolic and endosomal PRRs and trigger an inflammatory response. TLR9 is expressed on both the apical and basolateral sides of IECs (Figure 1). Ligand recognition on the apical side activates a tolerogenic effect, whereas stimulation of TLR9 on the basolateral side induces a robust inflammatory response (Lee et al., 2006). However, apical stimulation of TLRs primed by LPS leads to immediate degranulation and secretion of AMPs in Paneth cells (Yokoi et al., 2019).

IECs also have an overall muted response to LPS due to a low expression of TLR2, TLR4, the co-receptor MD-2, and CD14 (Abreu et al., 2001; Funda et al., 2001). Many of the TLRs are expressed and localized exclusively in the crypt epithelial cells of both the stomach and intestine (Cario et al., 2000; Kawai and Akira, 2008; Abreu, 2010), where they are inaccessible to commensal bacteria. Small intestinal IECs have been found to express very low levels of several TLRs that are normally highly expressed in the colonic IECs (Price et al., 2018). Furthermore, TLR4 has been reported to be sequestered in the Golgi apparatus (Hornef et al., 2002) and requires prior internalization of LPS to induce an immune response (Hornef et al., 2003).

There are other mechanisms that have been investigated to better understand commensal tolerance at the epithelial barrier. It has long been speculated that bacterial pathogens may modulate host epigenomics as a part of their virulence to establish contact during invasion. In the case of commensal microbes, the analysis of epigenetic modifications in the IECs of germ-free mice revealed a low level of methylation on the genes encoding for the LPS sensor TLR4, suggesting that commensal bacteria may be able to induce tolerance through epigenetic repression of genes encoding for PRRs (Takahashi et al., 2011). Commensal microbes have been shown to actively dampen the overall immune response to their MAMPs by suppressing the activation of pro-inflammatory pathways upon PRR sensing. In resting cells, NF-κB is sequestered in the cytoplasm by IκB, which masks NF-κB’s nuclear localization sequences. When the receptor is stimulated, classical NF-κB activation occurs by phosphorylating IκB, targeting it for ubiquitination and subsequent proteasomal degradation (Nigro et al., 2014a). Commensal bacteria have been shown to prevent the degradation of phosphorylated IκB by interfering with the host cellular machinery that controls the processes of ubiquitination and degradation (Neish et al., 2000; Collier Hyams et al., 2005; Tien et al., 2006).

A variety of inhibitors such as IRAK-M, Tollip, SIGIRR, and A20 regulate TLR and NLR responses in IECs (Shibolet and Podolsky, 2007). These inhibitors regulate the potential for chronic inflammation in the gut by dampening the response of the PRRs. SIGIRR has been characterized to play an active role specifically in the IECs (Shibolet and Podolsky, 2007). Inhibitors such as A20 have been studied to regulate NLRs in immune cells, but the same has not yet been demonstrated in IECs (Hitotsumatsu et al., 2008). miRNA-mediated regulation has also emerged as a central regulatory mechanism supporting tolerance to commensal MAMPs in the gut. In particular, MiR-155 plays an important role in attenuating *Helicobacter pylori*–induced inflammation in gastric epithelial cells (Xiao et al., 2009).

## 4 Epithelial recognition of microbial signals and its implications in diseases

### 4.1 Infection and inflammatory state

Despite an overall muted response to the presence of microbial cues and a tolerogenic response to MAMPs at the epithelial barrier, the primary function of IECs remain to be the identification and expulsion of any invading pathogen. The inflammasome-forming NLRC4 is a sensor of flagellin and bacterial secretion systems, and its epithelial expression promotes the extrusion of infected IECs from the epithelial layer (Nordlander et al., 2014; Sellin et al., 2014). NLRC4 has also been implicated for its role in protecting the host from intestinal carcinogenesis (Hu et al., 2010; Allam et al., 2015).

Interestingly, PRR signaling is tightly connected to mitochondria and the cellular response to microbial stimulation can be orchestrated at mitochondria. For example, it is well established that activation of various TLRs, including TLR1, TLR2, and TLR4, induce translocation of TRAF6 to mitochondria, resulting in increased ROS production, thereby promoting an inflammatory response (West et al., 2011). Interestingly, in addition to mitochondrial ROS production under pro-inflammatory conditions, mitochondria are also capable of releasing mitochondrial DNA, which acts as a DAMP, thereby promoting inflammatory pathways mediated by TLR9-dependent mechanisms. These pathways might play a role in the pathophysiology of chronic inflammation in humans, as mitochondrial DNA levels are significantly elevated in plasma samples of both UC and CD patients and correlate with disease severity (Boyapati et al., 2018). Pro-inflammatory mechanisms are also mediated by the binding of oxidized mitochondrial DNA to the NLRP3 inflammasome, which induces inflammasome activation during apoptosis (Shimada et al., 2012; Iyer et al., 2013).

Mouse models of ulcerative colitis (UC) are effective tools for studying inflammation in the context of diseases, and several models have successfully established the relationship between excessive NF-κB activation and the pathogenesis of IBD. However, several mouse models have also demonstrated a beneficial role for NF-κB (Wullaert et al., 2011) in maintaining intestinal homeostasis. NEMO is involved in the activation of the canonical NF-κB signaling pathway, and a mouse model with IEC-specific deletion of NEMO shows spontaneous development of severe chronic colitis, characterized by epithelial ulceration, infiltration of immune cells, increased expression of pro-inflammatory mediators, impaired expression of antimicrobial peptides, and translocation of bacteria into the bowel wall (Nenci et al., 2007). This is thought to be due to the fact that NF-κB deficiency leads to apoptosis of colonic epithelial cells, triggering a chronic inflammatory response in the colon. NEMO deficiency also sensitizes epithelial cells to tumor necrosis factor (TNF)-induced apoptosis, triggering inflammation even in the absence of NF-κB activation (Nenci et al., 2007). Mice lacking TAK1, a molecule that acts upstream of the IKK complex in IECs, have also been shown to develop spontaneous intestinal inflammation, supporting the role of NF-κB activation in maintaining intestinal mucosal homeostasis (Nenci et al., 2007). These phenomena suggest a double-edged function of inflammatory pathways in epithelial cells, as they both contribute to the maintenance of intestinal homeostasis and facilitate a rapid detection and clearance of pathogens upon invasion.

### 4.2 Inflammatory bowel disease (IBD)

Chronic inflammation in the gut is a causative factor in the pathogenesis of IBD, including Crohn’s disease and ulcerative colitis. Several factors related to PRR signaling at the epithelial barrier are involved in the induction and development of an inflammatory state in the gut.

Dysbiosis is strongly implicated in causing IBD. Exogenous introduction of an *Escherichia coli* strain associated with Crohn’s disease into TLR5 KO mice has been shown to promote disease pathogenesis (Carvalho et al., 2012), suggesting that immune dysfunction is an adjunct to specific microbial alterations in the development of IBD. A number of genetic aberrations, including NOD2 (Ogura et al., 2001; Macpherson and Uhr, 2004), which is associated with immune activation by peptidoglycans, and ATG16L1 (Hampe et al., 2007; Rioux et al., 2007), which plays a role in autophagy, have been implicated in the development of chronic inflammation. Variations in the TLR(s)- 2, −4, −5, and −9 genes have also been implicated in the pathogenesis of Crohn’s disease (Pierik et al., 2006; Toeroek et al., 2009; Eberl and Boneca, 2010; Chassaing et al., 2014). Epithelial-specific deletion of TLR5 has also been shown to result in microbial dysbiosis and low-grade chronic inflammation (Chassaing et al., 2014).

The individual role of TLR signaling in the development of gut inflammation may depend on the cell type and the interactions between individual TLRs. In the case of colitis development associated with TLR4 signaling, constitutively active TLR4 in epithelial cells did not induce mucosal inflammation in villin-TLR4 transgenic mice (Shang et al., 2008; Fukata et al., 2011). Although selective deletion of MyD88 in IECs results in spontaneous inflammation of the small intestine (Gong et al., 2010), suggesting a protective function of MyD88, MyD88 signaling in myeloid cells was found to be a driver of intestinal inflammation (Asquith et al., 2010).

One of the major factors contributing to the development of chronic inflammation in IBD is the loss of tolerance to commensal MAMPs at the epithelial barrier. Suppression of TLR9 signaling by adenoviral oligodeoxynucleotides has been shown to suppress intestinal inflammation in several mouse models of chronic colitis (Obermeier et al., 2005). Adenoviral oligodeoxynucleotides block the effect of bacterial cytosine-phosphate-guanosine oligodeoxynucleotides, and therefore innate immune signaling by commensal-derived DNA has been demonstrated as one of the factors inducing intestinal inflammation through activation of TLR9 during chronic colitis. Thus, while TLR signaling contributes to cytoprotection and mucosal restitution in the DSS colitis model, it may also be involved in promoting persistent mucosal inflammation in response to commensal bacteria (Obermeier et al., 2005).

NOD2 regulates the intestinal commensal flora through the secretion of bactericidal factors (Petnicki-Ocwieja et al., 2009). Impaired NOD2 signaling has been shown to alter commensal composition and increase susceptibility to intestinal inflammation due to defective secretion of antimicrobial peptides in the gut, followed by an abnormal immune response to the altered commensal flora (Petnicki-Ocwieja et al., 2009). Impairment of epithelial repair due to certain polymorphisms associated with TLRs is one of the factors contributing to the development and progression of IBD. TLR2 has been shown to play an important role in the induction of connexin-43 (Cx43)-mediated intracellular communication through intracellular gap junctions and controls IEC barrier function and restitution during acute and chronic inflammatory injury (Ey et al., 2009).

The nuclear receptor peroxisome proliferator-activated receptor-γ (PPAR-γ) regulates the expression of NLRP6 in IECs. PPAR-γ, in turn, is known to be induced by TLR4 signaling. This provides a potential anti-inflammatory role for TLR4 as it indirectly regulates NLRP6-mediated protection against DSS-induced mucosal damage (Dubuquoy et al., 2003; Kempster et al., 2011). Furthermore, NLRP3 has a prominent role in intestinal stromal cells in providing resistance to DSS-induced colitis. Both *Caspase-1* ^*−/−*^ and *NLRP3* ^*−/−*^ mice have impaired epithelial proliferation and increased mucosal permeability accompanied by defective healing responses to mucosal damage during DSS-induced colitis (Zaki et al., 2010a). However, the mechanism of NLRP3-mediated regulation of epithelial proliferation is still unknown, although it may involve IFN-γ and IL-18 in the regulation of cell proliferation (Fantuzzi et al., 1998; Nava et al., 2010; Zaki et al., 2011). Dectin-3 deficiency was also found to promote colitis development through severe colonic epithelial cell damage and impaired mucosal healing in the DSS colitis model. This suggests that CLRs may have additional roles in disease pathogenesis (Wang et al., 2016).

### 4.3 Colorectal carcinoma

Chronic intestinal inflammation triggers tissue transformation to become neoplastic and promotes a higher incidence of colorectal cancer in patients with IBD. Abnormal PRR signaling is thought to result in the dysregulated expression of genes and enzymes that regulate cell apoptosis, proliferation, and DNA repair. Frequent cycles of epithelial injury and repair, as in the case of chronic intestinal inflammation, in the presence of tumor-promoting cytokines, chemokines, and prostaglandins, may also act as a predisposition to genetic mutations, thereby increasing the risk of neoplastic transformation (Kundu and Surh, 2008; Ono, 2008). TLR4 stimulation has been shown to promote the proliferation of human IECs via epidermal growth factor receptor ligand expression (Hsu et al., 2010). Abnormal signaling via TLR2 and TLR4 in both IECs and sub-epithelial macrophages has been shown to induce dysregulated epithelial proliferation and therefore may promote the development of malignancies in the setting of chronic intestinal inflammation. Dysbiosis that arises in the absence of NLRP6 has also been demonstrated to promote cancer development through IL6-induced epithelial proliferation (Hu et al., 2013).

In the AOM-DSS-induced colitis-associated cancer model in mice, a single injection of azoxymethane (AOM) followed by repeated cycles of DSS treatment and periods of recovery is used to model colitis-associated cancer. The model represents recurrent mucosal injury and repair leading to dysplastic transformation in the colon (Suzuki et al., 2005). Some of the work in this mouse model provides a better understanding of the interplay between the various IEC-expressed TLRs in inducing epithelial hyperplasia under chronic inflammatory conditions. The *TLR4* ^*−/−*^ mouse has been shown to be protected against tumor development with reduced expression of mucosal COX-2, PGE2 and amphiregulin (Fukata et al., 2007). Bone marrow transplant-based analysis of the role of selective TLR4 signaling in colonic epithelial cells *versus* the myeloid cells showed a seminal role TLR4 signaling in epithelial cells in the development of dysplastic lesions (Fukata et al., 2009). This highlights the central role of innate immune signaling in epithelial cells in the formation of dysplastic lesions, as well as the recruitment of Cox-2-expressing macrophages and neutrophils during the development and progression of colorectal cancer. Conversely, *TLR2* ^*−/−*^ mice treated with AOM-DSS have been shown to have an increased tumor incidence with rampant proliferation and dampened apoptosis, although TLR2 deletion under normal conditions shows a reduced proliferation and increased apoptosis in IECs (Lowe et al., 2010). This increased tumor burden in *TLR2* ^*−/−*^ mice was further explained by the overactivation of signal transducer and activator of transcription 3 (STAT3) in epithelial cells and the elevated expression of tumor-promoting cytokines, such as IL-6, IL-17A and TNF-α in the gut mucosa.

*MyD88* ^*−/−*^ mouse models show variable responses to carcinoma challenge, depending on the differential inflammation in the models (Uronis et al., 2009; Salcedo et al., 2010; Schiechl et al., 2011). *MyD88* ^*−/−*^ mice show no proliferation in IECs after AOM-DSS treatment (Schiechl et al., 2011), however, *MyD88* ^*−/−*^ mice show an overall increased susceptibility to AOM-DSS induced intestinal tumors due to an upregulation of Wnt signaling associated genes, angiogenesis and DNA repair. *MyD88* ^*−/−*^ mice also show a higher mutation rate in the β-catenin gene in IECs as a result of AOM-DSS treatment, explaining the susceptibility to tumor pathogenesis (Salcedo et al., 2010). *MyD88* ^*−/−*^ mouse models further explain the development of tumorigenesis in the context of chronic inflammation. In the absence of a chronic inflammation, MyD88 deficiency has been shown to result in resistance to intestinal tumor development in the *Apc* ^*Min/+*^ and AOM mouse models (Rakoff-Nahoum and Medzhitov, 2007; Salcedo et al., 2010), demonstrating that MyD88 signaling can have both tumorigenic and anti-tumorigenic effects depending on the inflammatory context (Brandl et al., 2010).

The *NLRP3* ^*−/−*^ as well as the *NLRP6* ^*−/−*^ mice have been shown to have a higher incidence of intestinal tumors in the AOM-DSS model due to their inability to produce mature forms of IL-18 and IL-1β (Allen et al., 2010; Zaki et al., 2010b; Hu et al., 2010; Chen et al., 2011). Deletion of functional NLRC4, NLRP12, and caspase-1 also results in increased incidence of tumorigenesis in mouse models (Chen et al., 2008; Hu et al., 2010). The exact mechanism underlying increased IEC proliferation in NLR-deficient mice remains largely unknown, but *NLRP6* ^*−/−*^ mice have shown increased expression of proto-oncogenic genes such as *Mycl1* involved in the Wnt pathway in the AOM-DSS model (Normand et al., 2011), highlighting the possibility of similar mechanisms involved in the tumorigenesis in other knock-out models.

MUC2 deficiency in mice results in an increased predisposition to inflammation-induced colorectal cancer due to the inability to produce mucin via goblet cell stimulation (Velcich et al., 2002; Van-der Sluis et al., 2006). The specific role of epithelial cell signaling in the pathogenesis of colorectal cancer, independent of myeloid cells, is a very interesting facet of studying the immunogenic role of IECs in the gut. Deficiency of the epithelial cell-specific MyD88-dependent MMP7 molecule in the *Apc* ^*Min/+*^ mouse model of human familial adenomatous polyposis has been shown to reduce the incidence of tumorigenesis by more than 60% (Wilson et al., 1997; Rakoff-Nahoum and Medzhitov, 2007). Furthermore, MyD88-mediated tumorigenesis driven by epithelial cell signaling has been shown to result in the post-transcriptional stabilization of the c-myc protein, which is involved in the upregulation of anti-apoptotic mechanisms, proliferation and angiogenesis (Lee et al., 2010). This study using the *Apc* ^*Min/+*^ mouse model provides direct evidence of the IEC-dependent signaling pathway leading to rampant IEC proliferation and tumor growth.

The relative role of epithelial cells *versus* the myeloid cells in colitis-associated tumor development is further validated by the observed reduction in tumor incidence in IEC-specific IKK-β deletion, without affecting the overall intestinal inflammation of both AOM and *Apc* ^*Min/+*^ mouse models (Greten et al., 2004). NOD1 signaling has been shown to be protective against colon tumor development in both AOM-DSS and *Apc* ^*Min/+*^ models, where NOD1 plays a pivotal role in maintaining the intestinal epithelial barrier against chemically induced chronic injury, as in the case of these mouse models (Chen et al., 2008). In humans, NOD2 mutations have been associated with a significant risk of developing colorectal cancer (Mockelmann et al., 2009; Tian et al., 2010).

## 5 Summary

PRR-mediated microbial recognition and signaling at the intestinal epithelial barrier plays a multifaceted role in maintaining epithelial barrier function and homeostasis, microbial composition and localization, development of overall mucosal immune functions, and defines a number of host physiological and metabolic functions. Several mouse model-based studies using IEC-specific PRR knockouts have demonstrated the importance of PRR signaling at the epithelial barrier and its impact on the various immune and metabolic functions in this region. However, further investigation is required to characterize the expression and specific roles of a number of these PRRs in IECs, independent of their roles in myeloid cells. Recently, the β-glucan receptor Dectin-1 has been shown to be a positive inducer of intestinal prostaglandin E2 (PGE2) secretion by myeloid-derived suppressor cells (MDSCs), leading to enhanced colorectal tumorigenesis in human colorectal cancer patient cohorts as well as in AOM-DSS and *Apc* ^*Min/+*^ mouse models. Dectin-1 signaling was correlated with increased PGE2-synthase expression and suppressed *IL22RA2* in human CRC-infiltrating cells (Tang et al., 2023). The intestinal epithelial barrier is the primary site of CRC disease pathogenesis and progression, and thus it is intriguing to question the exact role of Dectin-1 signaling, among other innate immune signaling in IECs in CRC development and pathogenesis. Several innate immune receptors and the resulting crosstalk between the drivers of these signaling cascades have so far only been characterized in myeloid cells, and their independent roles on IECs warrants further investigation. However, in the above-mentioned study, *Tang et al.* could not specifically determine the effect of Dectin-1 receptor ablation on PGE2, as well as *Il22ra2* expression in the intestinal epithelial cells (Tang et al., 2023).

Innate immune signaling at the epithelial barrier has a complex role due to its proximity to the gut microbiota. Although dysbiosis, or a shift in the overall microbial population at the mucosal region, has been implicated in the pathogenesis of several inflammatory diseases as discussed in this review, there is no consensus on the exact composition of the microbiota in health *versus* disease (McBurney et al., 2019). In addition, it would be critical to understand the multitude of factors that lead to the loss of an overall tolerogenic immune response in the mucosal region, leading up to a chronic inflammatory state. Dysbiosis, as a pathologically relevant factor in intestinal inflammatory disorders, is further hampered by the limitations in understanding weather a change in the commensal microbial population is a prerequisite for the development of inflammation or whether the onset of an inflammatory setting leads to a shift in the microbial population. Furthermore, the labeling of certain commensals involved in the development of inflammatory diseases as pathobionts has been questioned and the inclusion of additional factors such as the ‘microbial context’ has been emphasized to play a key role in defining the opportunistic properties of the gut microbiota in inflammation (Jochum and Stecher, 2020). A deeper understanding of the role of different commensal strains in different contexts of infection and inflammation would allow a broader definition of their contribution to disease pathogenesis.

Crosstalk between the various drivers of innate immune signaling pathways is another very interesting component of PRR signaling at the epithelial barrier. The mechanistic activation of IEC-bound PRRs by their cognate ligands and how activation of these PRRs influence the various canonical and non-canonical signaling pathways requires further investigation. In this context, an orphan nuclear receptor, Nur77, has recently been shown to sense intracellular LPS, leading to non-canonical NLRP3 inflammasome activation via gasdermin-D (GSDMD) processing in macrophages (Zhu et al., 2023). Nur77 expression in macrophages was shown to increase upon treatment with various cognate TLR ligands, suggesting a possible crosstalk between the canonical and non-canonical signaling pathways of LPS sensing. Further studies investigating such convergent signaling pathways would highlight such crosstalk and their net physiological impact on innate immune signaling in IECs.
